# TreeViewJ: An application for viewing and analyzing phylogenetic trees

**DOI:** 10.1186/1751-0473-2-7

**Published:** 2007-10-31

**Authors:** Matthew W Peterson, Marc E Colosimo

**Affiliations:** 1 The MITRE Corporation, 202 Burlington Rd, Bedford, MA, USA; 2 Department of Biomedical Engineering, Boston University, 44 Cummington St, Boston, MA, USA

## Abstract

**Background:**

Phylogenetic trees are widely used to visualize evolutionary relationships between different organisms or samples of the same organism. There exists a variety of both free and commercial tree visualization software available, but limitations in these programs often require researchers to use multiple programs for analysis, annotation, and the production of publication-ready images.

**Results:**

We present TreeViewJ, a Java tool for visualizing, editing and analyzing phylogenetic trees. The software allows researchers to color and change the width of branches that they wish to highlight, and add names to nodes. If collection dates are available for taxa, the software can map them onto a timeline, and sort the tree in ascending or descending date order.

**Conclusion:**

TreeViewJ is a tool for researchers to visualize, edit, "decorate," and produce publication-ready images of phylogenetic trees. It is open-source, and released under an GPL license, and available at .

## Background

Phylogenetic trees are widely used to visualize evolutionary relationships between different organisms or samples of the same organism. There is a variety of both free and commercial tree visualization software [1-5] available, but limitations in these programs often require the user to use multiple programs for analysis, annotation, and display for publication. For some applications, such as epidemiological studies, the visualization of sample collection dates along with the tree would provide further insight into relationships. However, no currently available visualization packages provide this functionality.

A variety of file formats exist for the storage of phylogenetic trees. Some, like the New Hampshire[6] format store only the node name and branch length information. Others, like the New Hampshire Extended [5] and Nexus[7] formats, can be used to store other data such as formatting and sequence data. Other file formats, such as the Lucid [8] and Structure of Descriptive Data (SDD) [9] formats have been used for the description of taxonomic data. Recently, a draft standard for an XML-based format, PhyloXML [10], has been released. XML-based formats are ideal for storing phylogenetic data because they can be easily interpreted by readily available general purpose software (thus eliminating the need for customized parsers), easily allow for annotation, and are extensible.

We have designed a visualization program, TreeViewJ, which will allow researchers to annotate, edit and produce publication-ready figures of phylogenetic trees, and to visualize sample collection date information using a timeline. With the use of PhyloXML, there is more flexibility for editing trees – trees can be edited before visualization with an XML parser, as well as within the tool. We have also implemented an algorithm for sorting taxa by date, while keeping the structure of the tree the same.

## Implementation

TreeViewJ is written in Java, against JDK 1.5. The Swing and Abstract Windowing Toolkit (AWT) libraries are used for the graphical user interface, and the Java2D libraries are used for drawing tasks. This allows the application to be platform independent. While it is meant to be a standalone application, the object-oriented design of TreeViewJ allows straightforward integration with other Java applets or applications.

## Results and discussion

### Graphical User Interface

**Figure 1:**
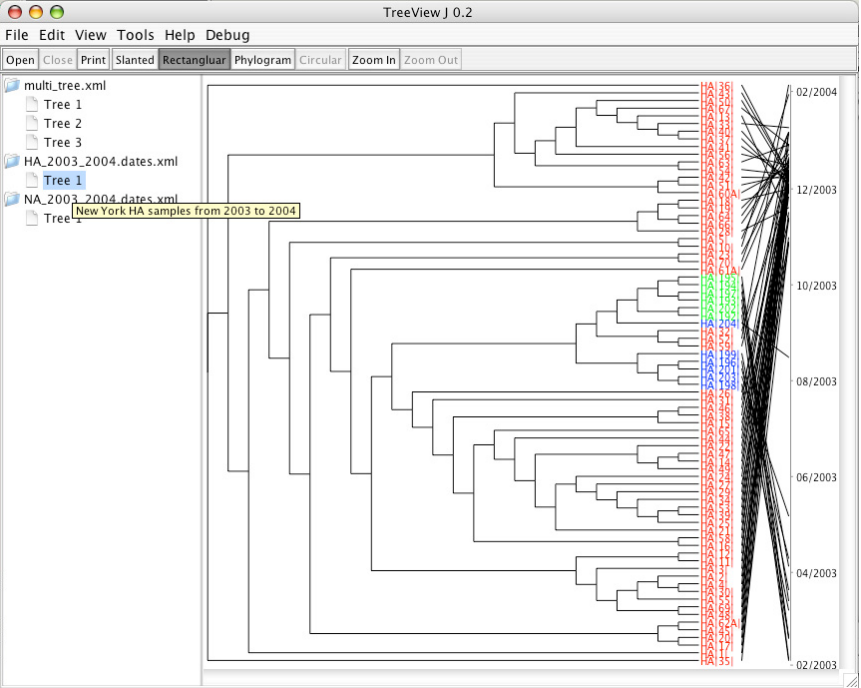
**TreeViewJ User Interface**. The main user interface for TreeViewJ. Multiple files, one of which contains multiple trees, are open. The currently displayed tree contains colored taxon names, and has had its taxa mapped to a timeline.

The main user interface for TreeViewJ, shown in Figure 1, is composed of two panels. The left panel displays open files (referred to as "forests") and the trees stored within them. Tooltips are used to display a description of the tree, if one exists. The user can move trees between files by either copying and pasting, or using drag and drop. Trees may also be deleted from forests. Visualization and editing options are easily accessible via menus and toolbars. The right panel contains a scroll pane which displays the currently selected tree.

### File Input and Output

TreeViewJ offers full read support for Newick and PhyloXML formatted files. Trees in Nexus [7] and Extended Newick [5] format can be imported, though some information contained in these files may be lost. Currently, there is no support for Lucid or SDD file formats, but these could easily be added in the future. PhyloXML files are read using the Apache Xerces Document Object Model (DOM) parser [11]. Newick files are parsed using code adapted from the Phylogenetic Analysis Library (PAL) [12], and Nexus files are parsed with a custom parser. Files are written to XML documents using Xerces.

Each file may contain multiple phylogenies. Forests may be saved to PhyloXML format, and individual trees can be exported to Newick format.

### Visualization

TreeViewJ can represent phylogenies as slanted cladograms, rectangular cladograms, circular cladograms, and phylograms. The tree drawing code was adapted from TreeViewX [4], an open source project based on TreeView [3]. Trees are drawn using the Java2D libraries.

Once a tree is drawn the user can zoom in and out, change the draw color, and toggle internal and leaf labels on and off. Zooming and resizing are achieved using affine transforms to avoid redraws. Nodes of the tree can be selected by right clicking or clicking and dragging and their labels may be copied to the clipboard and pasted into other applications.

Once the tree is drawn, it can be printed or exported to either Scalable Vector Graphics (SVG) or JPEG format. The Apache Batik [13] toolkit is used to create SVG documents.

### Editing

Node editing is started by clicking on one of the nodes in the tree. At this point, the node editing dialog is shown. The editor allows changes to be made to node name and font color, as well as the branch width and color. Possible uses for this functionality would be easily highlighting parts of the tree that are described in the text, or adding label to the parent node of a clade. Once editing is completed, the changes are made to the XML document and the tree is refreshed to display the changes made. Nodes can also be searched for using Java regular expressions or plain string mapping.

### Date Mapping

Currently, the PhyloXML standard does not provide any way to store date information. We have proposed an addition to the schema which allows for the addition of sample dates, using the ISO 8601 date format. Dates stored in the XML document as such are mapped to the leaf nodes using a timeline, as shown in Figure 1. The JFreeChart [14] library, along with some additional code, was used to create timelines. Java2D libraries were used to draw lines from the leaf nodes to their dates. Dates are mapped at a minimum resolution. For example, if the dates span less than two years, dates are displayed as mm/yyyy. If the dates span less than a year, then the dates are displayed as mm/dd/yyyy.

### Date Sorting

**Figure 2:**
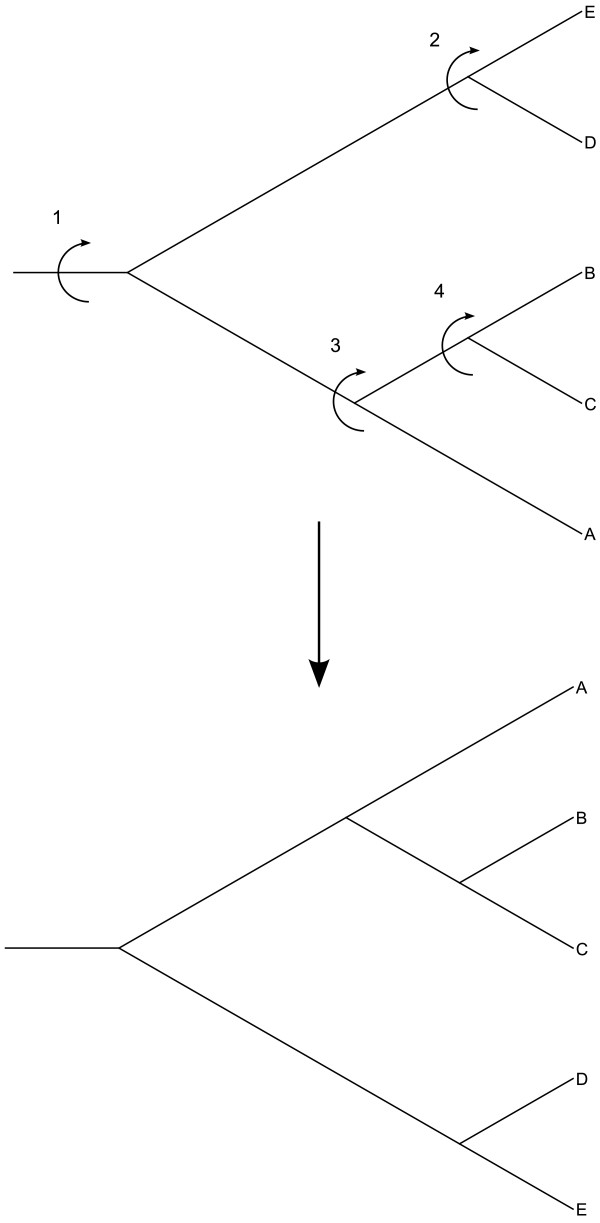
**Tree Sorting in TreeViewJ**. The sorting algorithm used in TreeViewJ. Here, it is being used to sort the taxa alphabetically. The numbers above the internal nodes show the order that sorting is done in.

Phylogenic trees are sorted by date in the most straightforward way possible: a depth-first post-order sort of the children at each node. Leaf nodes are assigned the dates of the samples they represent. Internal nodes are assigned the minimum (ascending) or maximum (descending) date of its children. The resulting tree is isomorphic to the original tree, with the taxa sorted in ascending (oldest samples on top) or descending (youngest on top) order (Figure 2).

## Conclusion

We present TreeViewJ, an easy to install, easy to use, open source tool for visualizing and editing phylogenetic trees. It is written in Java, and therefore is platform independent. It is the first tree viewer to include support for date mapping and sorting. The ability to draw, edit, analyze, and produce publication-ready vector graphics makes TreeViewJ an ideal tool for phylogenetic visualization, and an excellent complement to full-featured phylogenetic suites such as Mesquite [15] and PAUP [16].

## Availability and requirements

• **Project Name: **TreeViewJ

• **Project Home Page: **

• **Operating System**: Platform Independent

• **Programming Language**: Java

• **Other Requirements**: Java Runtime 1.5 or greater, Apache Maven

• **License**: GPL

• **Any restrictions to use by non-academics: **None

## Competing interests

The author(s) declare that they have no competing interests.

## Authors' contributions

Both authors contributed towards the source code, manuscript, and documentation. Both authors read and approved the final manuscript.
